# Development of a growth monitoring and promotion index to improve child health in Zimbabwe

**DOI:** 10.1016/j.mex.2022.101958

**Published:** 2022-12-11

**Authors:** Shamiso Alice Moyo, Ntsieni S Mashau, Lufuno Makhado

**Affiliations:** Department of Public Health, Faculty of Health Sciences, University of Venda, Private Bag x 5050, Thohoyandou, 0950 Limpopo Province, South Africa

**Keywords:** Caregivers, Community health workers, Barrier analysis, Children under five years, Index development, Zimbabwe, Sequential Explanatory Mixed Method

## Abstract

In Zimbabwe, growth monitoring and promotion as conducted by community health workers are part of the nutritional surveillance system. This study seeks to develop a new index which will combine both caregiver behaviours, attitudes and CHW growth monitoring and promotion activities. An explanatory sequential mixed method design will be conducted in three phases. Phase one will comprise a scoping literature review. The second phase will comprise a needs analysis through quantitative data collection using two surveys of community health workers and caregivers of children under five years. Thereafter, qualitative data will be collected from caregivers of children under five years. The quantitative data will be analysed using SPSS while qualitative data will be collected and analysed using Atlas-ti. Phase three will be the development phase for the growth monitoring and promotion Index. The growth monitoring and promotion Index will be used to classify the GMP performance of districts through the DHIS2 thus strengthening the quality of growth monitoring and promotion. Recommendations on the findings and the adoption of the Index will be shared with the Ministry of Health and Child Care and key stakeholders implementing maternal, newborn and child health programmes in Zimbabwe for adoption and use in growth monitoring and promotion programming.

## Graphical abstract

**Figure:**
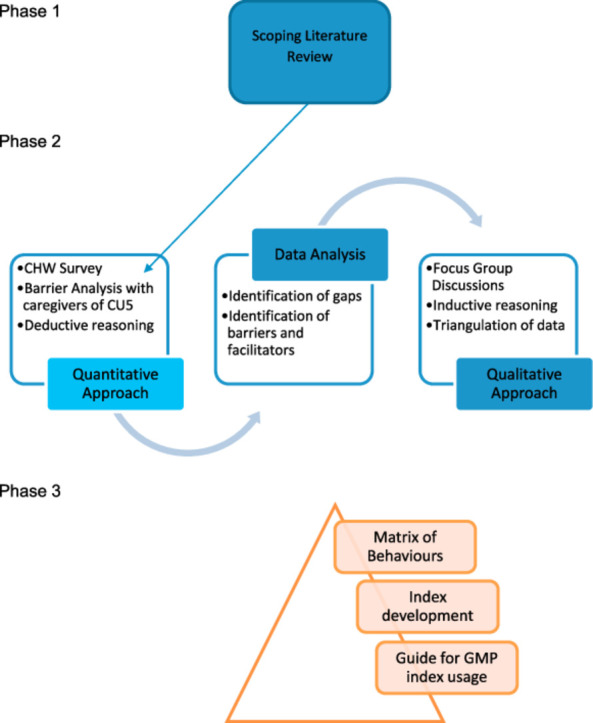

**Specifications table**

**Table:** 

**Subject area**	Medicine and Dentistry
**More specific subject area**	*Child Health*
**Name of your protocol**	*Development of a Growth Monitoring and Promotion Index to improve Child health in Zimbabwe*
**Reagents/tools**	*NA*
**Experimental design**	*NA*
**Trial registration**	*N/A*
**Ethics**	***If your work involved human subjects,****please include a statement here confirming that the relevant informed consent was obtained from those subjects:* Written Informed consent was sought from the caregivers of children under five years and Community Health Workers
**Value of the Protocol**	• *Describes in detail the development of a novel child health Growth Monitoring and Promotion Index* • *Proposing positive addition to the DHIS2 to classify Growth Monitoring and Promotion appropriately* • *Correct government financial resource allocation based on Growth Monitoring and Promotion Index classification*

## Description of protocol

### Background Information

In Zimbabwe, Growth Monitoring and Promotion (GMP) is part of the nutritional surveillance system which aims to serve as an early warning system for child growth and development problems. The community-based growth monitoring programme is carried out at the village level by CHWs who go on to report the number of children they monitor including those malnourished to the health facility within their catchment area. From the health facility, this data filters up to the district as part of the district health information system (DHIS2). Through this study, a GMP index will be developed to improve the quality of GMP activities to support the overall child health and nutrition of CU5 in the Umguza district.

### Study rationale

The classification of GMP, whether poor, good and so on is being currently done based on independent indicators in the DHIS2 such as the number of CU5 weighed monthly by Village Health Workers and the number of children under five (CU5) who received vitamin A. There is no assessment of why caregivers are not bringing their children for monthly GMP. This has resulted in a disconnect between caregiver behaviours and attitudes and the quantitative indicators in the DHIS2. For a more informed classification, all key indicators must be interconnected to each other and be part of an overall picture that considers the different elements of the GMP processes which is more meaningful and informative. The key elements of GMP being the CHWs activities and the behavior and attitudes of caregivers of CU5. The construction of the GMP index will ensure that all these GMP elements are represented in one informative Index in which caregiver behaviours, attitudes and CHW GMP activities are combined. This then provides more accurate information on the status of GMP activities in a particular district allowing for appropriate resource allocation based on accurate performance.

### Study purpose and objectives

The following are the study purpose and objectives:

#### Study purpose

To develop a GMP index to improve the quality of growth monitoring and promotion activities in the Umguza district.

#### Study objectives

***Phase 1: Literature review***

1. To explore and analyse knowledge gaps about GMP activities,
2. To describe key characteristics related to GMP index development.

***Phase 2: Needs analysis***

1) To explore the process of GMP within the Umguza health system as conducted by CHWs,
2) To determine how knowledge of growth monitoring and promotion by CHWs translates to a frequency of activities,
3) To determine the barriers and facilitators towards GMP attendance by caregivers of CU5,
4) To identify sustainable best practices among CHWs consistently conducting growth monitoring and promotion activities.

***Phase 3: Index development***

1) To construct a GMP Index and guidelines for usage,
2) To test and validate the GMP index.

#### Research questions

1) What is the process of GMP as conducted by CHWs?
2) How does knowledge of GMP by CHWs affect the frequency of activities?
3) What are the best practices among those CHWs consistently conducting GMP activities?
4) What are the barriers and facilitators towards GMP activities by caregivers of CU5?

### Conceptual framework for the study

The study will adapt the logic framework for GMP by Ashworth et al. [2]. This framework outlines the whole GMP process as would be conducted by a CHW or a health worker. This framework is underpinned by the benefits of GMP to the children which were classified as being mainly due to two mechanisms: first is the detection of the problem by the CHWs and the second being a vehicle for health promotion through positive behavior change adoption by caregivers of CU5 [2].

From this model, GMP activities are meant to be done by CHWs routinely within the MOHCC health system. These activities consist of weight, height and MUAC measurements and plotting into the child's health card and age-appropriate counselling for the caregivers. However, GMP at the community level is directly linked to the caregiver behaviours and attitudes towards it and the performance of CHWs in conducting the actual GMP activities. These are the constructs from which data will be collected.

This framework highlights two important players central to the GMP of CU5 which are the CHWs and caregivers. The study will focus on them both in terms of the current situation along with determinants of why this is so. The CHWs are the first point of contact with the caregivers hence it will be established through a survey of how they conduct the monthly GMP processes and activities in comparison to the recommended practices by MOHCC. One of the outcomes of this survey would be information that would improve the early detection and treatment of illness through increased GMP attendance. Through a barrier analysis with the caregivers of CU5, the study will explore the barriers and facilitators towards their GMP attendance. The outcome of this knowledge would aid in the identification of the different types of caregivers behaviours along with the specific social behavior change (SBC) strategies to meet their different needs.

Once caregiver behaviours and attitudes towards GMP have been identified and GMP activities by CHWs established, a GMP Index will be constructed. The researcher anticipates that due to the composite nature of the index, where it will consider practical caregiver behaviours and attitudes towards GMP and GMP activities being done or not done at the community level by CHWs, a more appropriate classification of GMP activities within the district may be done. The hope is that this would ultimately result in the design of a more targeted approach towards improved GMP activities and child health in general.

### Definition of concepts

**Child health:** Child health is defined by the National Research Council and Institute of Medicine (200: 4) as ‘the extent to which individual children or groups of children are able or enabled to (a) develop and realise their potential, (b) satisfy their needs and (c) develop the capacities that allow them to interact successfully with their biological, physical and social environments.

**Community Health Worker:** Community Health Worker is defined by Lewin et al. [[17]:7] as “any health worker carrying out functions related to health care delivery; trained in some way in the context of the intervention, and having no formal professional or paraprofessional certificate or degree in tertiary education”. In this study, the CHW being referred to is the Village Health Worker (VHW).

**Health Care Worker**: The term “health care worker” has previously been defined as “all people engaged in actions whose primary intent is to enhance health” and was not restricted to, physicians, nurses, allied health personnel, health educators, social workers, midwives, community health workers, laboratory personnel, pharmacists, radiographers, volunteers, orderlies, and health-facility administrators [14], [35]. In this study, the HCWs being referred to are doctors, nurses, environmental health technicians and district nutritionists.

**Growth monitoring:** Growth monitoring is the regular weighing, measuring and plotting of a child's growth over time to carry out scientific interpretations that aid in counselling, while also creating evidence-based interventions if abnormal growth is identified [10]. This study will make use of the definition below.

**Growth monitoring and promotion:** According to WHO guidelines, Growth Monitoring and Promotion includes (1) the routine measurement of a child's weight and length/height; (2) the plotting of the child's measurements and comparison of the child's status to a standardised growth chart to assess growth adequacy; (3) growth‐informed counselling; and, if necessary, (4) the undertaking of remedial, health‐promoting action [35]. This study will refer to this definition as defined by WHO.

**Growth monitoring and promotion index:** A composite Index derived from the metrics of the barriers and facilitators towards GMP by caregivers of CU5 years and the monthly GMP statistics of CU5 years as conducted by CHWs. This index is based on the GMP definition stated above.

**Primary Health Care Facility:** This is the most basic structured health system providing care for simple, common problems at the community level, [3] In this study, this refers to clinics found in the district.

**Caregivers of CU5:** Caregivers will be the mothers or other family members who are primarily responsible for attending to the child's health [20]. This will refer to mothers or legal guardians who take care of the children under five years living in the Umguza district.

**Nutritional Surveillance:** This is the regular and systematic data collection on nutritional outcomes and exposures, [31].

## Methodology

The study will follow a mixed-methods approach.

### Research approach

A mixed-methods research approach is where the researcher combines both quantitative and qualitative research approaches to increase the breadth and depth of understanding of the study elements [9]. In this study, both quantitative and qualitative data will be collected sequentially. The researcher chose a mixed-method approach so as to enhance the integrity of the findings, to obtain generalisability of the results along with depth that will come from the results [9]. The mixed-methods approach will also provide complementarity of the results as each method seeks to address different objectives of the study.

#### Outline of the study process

The study will be conducted in three phases. The first phase will involve a scoping literature review. The second phase will begin with the collection of quantitative data through a survey of CHWs and a barrier analysis of caregivers of CU5. Data analysis will then be done and gaps for further probing identified. Qualitative data will then be collected through focus group interviews and analysed. All the findings will then be triangulated. The third phase will involve the development of an Index to measure GMP through CHW GMP activities and CU5 caregiver behaviours towards GMP. The study will also come up with guidelines on the use of the index. An illustration of the study phases is shown in Fig. 2.

### PHASE 1

This phase will comprise a scoping literature review. This review will target both quantitative and qualitative studies the world over up to December 2021 on GMP activities. The studies may be in the form of published articles in peer-reviewed journals or original research and reports found on Google Scholar, Science Direct, PUBMED and EBSCO. A search criterion using selected keywords will be used to streamline relevant studies.

#### Design

A scoping literature review will be done. This is so as to explore the breadth or extent of the available literature, map the evidence and inform future research [29]. In this study, the breadth and extent of available literature on GMP activities will be explored along with any GMP indexes that have been developed and their contribution to child health.

#### Review title

GMP activities and the development of a GMP index for improved child health: A scoping review.

#### Review questions

1. What are the gaps for further research on GMP activities?
2. What indexes have been developed towards improving child health through GMP?

#### Specific objectives

1. To explore and analyse knowledge gaps about GMP activities,
2. To describe key characteristics related to GMP index development.

#### Search strategy for identification of studies

Keywords aligned to the study objectives will be used to search for relevant literature from Science Direct, Google Scholar, EBSCO and PUBMED databases. Reference lists of identified articles will be searched for additional sources.

#### Inclusion criteria

Based on the definition of GMP, the scoping literature review will consider all published studies and reports about GMP activities at the community level as conducted by CHWs. The GMP activities are for children under 5 years of age. These studies would have been published in peer-reviewed journals the world over up to December 2021 along with unpublished (grey literature) found up to December 2021. The review will also target both quantitative and qualitative research and reports from Science Direct, Google Scholar, EBSCO and PUBMED databases.

#### Exclusion criteria

This scoping literature review will exclude GMP activities done at the primary health facility level along with any other GMP activities not conducted by CHWs or equivalent cadre within the health system.

#### Study selection

Study titles and abstracts will be reviewed independently by at least two reviewers to identify and compare studies and reports relevant to this scoping review. Once defined as relevant, the full articles will be reviewed. Any disagreements will be solved by consensus. A detailed flow chart using the PRISMA-ScR guidelines will be made to show how articles were selected.

#### Data extraction/data charting

A data collection form will be developed which is guided by Rodgers’ Evolutionary Conceptual Analysis Framework [27], to ensure uniformity and quality in the extraction of data from studies that meet the inclusion criteria by reviewers. Through the use of this framework, aspects such as the study concepts, context, antecedents, attributes and consequences would be assessed. The strength of this framework is that it can contribute to clarifying, describing and explaining concepts by analysing how a chosen concept has been used within the discipline [28].

#### Analysis of the evidence

The results will be descriptively mapped. Each reviewer will use the data collection form and the results compared and agreed upon. The Rodgers Evolutionary Conceptual Analysis Framework will be used to assess the clarity results regarding further research on GMP activities at the community level and the indexes that have been developed towards improved child health.

#### Presentation of the results

The data will be presented in a chart. The ultimate purpose of charting the data will be to identify, characterise and summarise the research evidence and answer the scoping review questions [23].

### PHASE 2

The following are entailed in this phase:

#### Study design

The design will be an explanatory sequential mixed method. Using the explanatory sequential mixed-methods design, the researcher intends to first collect quantitative data, conduct data analysis, and identify gaps to be further investigated through a qualitative approach [8].

#### Study setting or area of study

The study will take place in the Umguza district which lies in Matabeleland North Province of Zimbabwe, in the south-western part of the country. The district shares its boundary with the city of Bulawayo, the second capital of Zimbabwe. The district has 19 wards, 25 primary health care facilities and 186 CHWs. According to the 2012 census, the district had a population of 89,687 people and of these, 52,5% were male while 47,5% were female. Of this population, the children aged 0 to 59 months are 12,549. The district also had a crude birth rate of 24 and an infant mortality rate of 62 [37]. According to the national nutrition survey of 2018, the prevalence rate for stunting is 26,9%, wasting is 3,75%, underweight is 9,1% and overweight is at 1,3%.

The population is mostly rural with a very small urban component and most people are engaged in agriculture-related occupations and cattle rearing as livelihood activities as the district greatly experiences dry weather conditions. Most of the households are headed by men and the average household size is 4.7 people [37]. The culture is predominantly Ndebele.

The district has been selected because according to the DHIS2 in the past 12 months, over 95% of the children under five years are not being taken for GMP monthly. This further indicates that the caregivers of these CU5 who are not attending GMP are being missed with regards to appropriate health education and counselling relevant to the child's particular health condition.

#### Study population and sampling

##### Target population

The target population for this study will be all the 186 CHWs in the district and 90 caregivers of CU5 as per standard Barrier Analysis methodology [15], [19]. The CHWs are volunteers who work within the MOHCC health system and coordinate all activities related to health at the village level and are conduits into the formal clinic-based care, [18]. The caregivers of children under five years will either be birth mothers or legal guardians of the children under five years living in Umguza district and who also live with and take care of the children.

#### Sample and sampling

##### Quantitative data

A census approach will be used, where all the CHWs from the 19 wards will be included. The use of a census approach ensures that all CHWs in the Umguza district are interviewed thus providing accurate information on GMP activities as conducted by all CHWs in the district [34]. The advantage of using a census approach is that all the CHWs will be interviewed and the results will be indicative of the actual activities and experiences of CHWs in relation to GMP in the Umguza district. Since the population size for the CHWs in the Umguza district is 186 [19], this will also translate to be the sample size of CHWs. All health facilities in the Umguza district have lists of CHWs and these will be used to identify CHWs and ensure that they will all be interviewed.

The second survey will be among the caregivers of CU5. This survey will follow the Barrier Analysis approach. A barrier analysis is a survey that focuses on identifying what is preventing the target population i.e., caregivers of CU5 from adopting the behaviour under investigation [15]. The sample size is given as 90 caregivers where there will be 45 individual doers (those who attend GMP monthly) and 45 non-doers (those who do not attend GMP monthly). Since there are 19 wards in Umguza, and 90 caregivers required, an equal number of caregivers per ward will be determined. Thereafter, using GMP registers for the CU5 kept by the CHWs, the caregivers will be selected randomly per ward and appointments made with them through the CHW for data collection at their homes.

##### Qualitative data

Homogenous, purposive sampling will be used to obtain the respondents who are the caregivers of CU5 [24]. It is stated by Gray [11] that the purpose of purposive homogenous sampling is to describe small homogenous groups in-depth. In this study, the groups will be the caregivers of CU5 who have not attended growth monitoring at all six months prior to the data collection.

A total of ten focus-group discussions (FGDs) with caregivers of children under five years will be conducted. These will be from purposively selected wards based on the poor performance of GMP statistics according to the DHIS and Barrier analysis survey. Boddy [4] states that samples in qualitative research can be small and dependent on the context. If the data is properly analysed, a saturation point will be reached when little or no new evidence will be obtained from the discussions. It is at this point that a larger sample size ceases to contribute to new evidence. If, however, data saturation has not occurred after interviewing the caregivers then, further respondents will be purposively selected.

#### Inclusion/exclusion criteria

##### Quantitative data collection

All the CHWs who reside and volunteer in the Umguza district will be included in the study, whilst 90 caregivers of CU5 will be selected for the Barrier analysis survey as well. The 90 caregivers will include 45 caregivers who attend GMP monthly and 45 who do not attend GMP monthly.

##### Qualitative data collection

The caregivers of CU5 who do not attend GMP monthly and who have not attended consistently for six months prior to the study will be included in the study.

Excluded will be other types of community volunteers such as home bases caregivers and community-based facilitators within the Umguza district along with caregivers who have no children less than five years.

#### Measurement instruments

##### Quantitative data collection

**Fig. 1:**
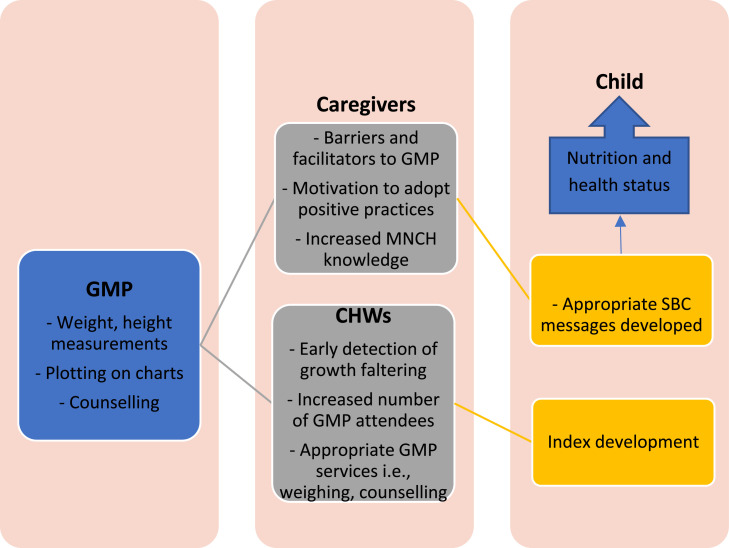
Conceptual framework for improved growth monitoring and promotion. Adapted from: Ashworth, Shrimpton & Jamil, [2].

Structured questionnaires with closed-ended questions will be used to collect survey data with the CHWs and caregivers of CU5. According to Polit and Beck [26], closed-ended questions ensure the comparability of responses among participants and facilitate data analysis. The researcher will develop the questionnaires which will be informed by the scoping literature review, the study objectives and the conceptual framework Fig. 1 by Ashworth et al. [2] which is overall guiding the study. This will be done by the researcher with guidance from the academic supervisors. The questionnaire sections will include demographical characteristics of the population, GMP activities conducted by CHWs, Knowledge of GMP activities by CHWs and Barriers and facilitators to caregiver GMP attendance. The questionnaires will be translated from English into Ndebele by a professional translator.

**Fig. 2:**
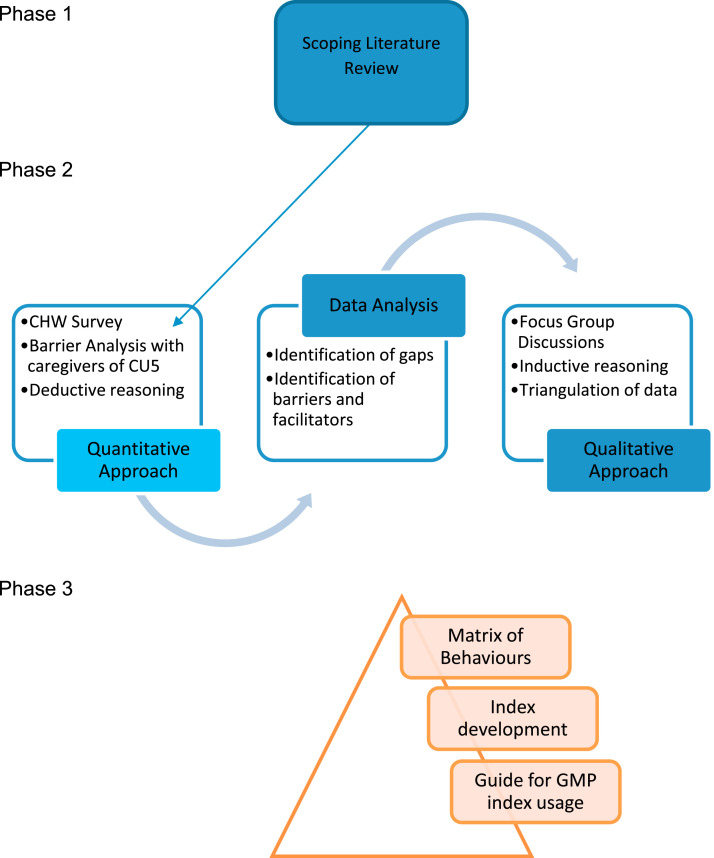
Diagrammatic illustration of the research approach.

Appointments with the CHWs and caregivers of CU5 will be set prior to data collection dates through the local health facility. Data collection will take place within the villages where the respondents reside.

##### Qualitative data collection

A semi-structured FGD interview guide will be used to facilitate the interviews with caregivers of CU5. The questions on these interview guides will be open-ended to allow for rich discussions. The questions in the guide will be developed after quantitative data analysis and the identification of any gaps or areas in need of further probing. FGD interviews will allow the researcher to gain deeper insight into the identified gaps regarding GMP activities as conducted by CHWs and influences of growth monitoring attendance by caregivers.

#### Pre-test

The pre-testing of the data collection tools will be done in Gwanda district, a different district that has similar characteristics in terms of poor performance in GMP according to the DHIS2. A total of ten CHWs and ten caregivers of CU5 will be interviewed to test for language and content suitability and make any corrections to the tools, note language translation suitability and take note of the possible duration of the questionnaire and in-depth interviews as well. All data collection tools will be translated into the local language of isiNdebele by a professional translator.

#### Validity and reliability

The following aspects will be considered in this study:

Validity of questionnaire

Validity is the degree to which an instrument measures what it is supposed to measure [25]. Using experts in the field, the study will use face validity. This will be done to measure the appropriateness of the questionnaire content by evaluating its appearance in terms of relevance to the construct, language clarity and readability and formatting consistency [30]. Revalidation of the tool will then be achieved through pre-testing the tools with the CHWs and caregivers of CU5. For revalidation, the questionnaires have to be confirmed by the survey respondents to be clear, understandable, easy to follow, with a consistent format and layout [36].

Reliability of questionnaire

The reliability of a questionnaire refers to the consistency with which participants understand, interpret, and respond to all questions in the questionnaire [5]. The questionnaires will be reviewed to determine the reproducibility or repeatability and internal consistency of their constructs. The Cronbach alpha correlation coefficient will be used to assess the internal consistency reliability of the questionnaires and for this study, the value for alpha should be greater or equal to 0.8 [30].

#### Plan for data collection

Quantitative data will be collected first and analysed. Thereafter qualitative data will be collected to address any further gaps. Data collection will take place in the villages where the caregivers reside. Appointments will be set prior to the FGDs through CHWs. Electronic recording devices will be used to record the discussions. The recording will allow the researcher to devote their full attention to listening and probing in-depth. The researcher is also able to provide an accurate account of the interview capturing the participants’ language and tone through field notes. Field notes are descriptive written in-depth accounts of happenings and experiences of the researcher during the research [16]. Polit and Beck [25] also indicate that field notes can contain summarised highlights of the discussion between the researcher and the participant. All the data will be transcribed into a notebook and then translated into English. Thereafter, the data will be typed into a Microsoft word document.

COVID-19 considerations will be taken to minimise the risk of exposure to and infection from Covid. The researcher will abide by the Zimbabwe MOHCC Covid-19 guidelines that will be existent at the time of the data collection. The researcher will ensure that all interviews are conducted in an open space that is well ventilated. Hands will be sanitized before the interview, while during interviews face masks will be worn always. A distance of at least 1,5 metres will be kept between the research participant and the researcher.

#### Two-day training for research assistants

The researcher will use two research assistants in the quantitative data collection process. A two-day training will be held to train on the study concepts, questions in the questionnaires, how to conduct good interviews and who the respondents are. Ideally, the research assistants should have some prior training in research ethics as well. All research assistants should be able to speak the local language of isiNdebele. The training will be conducted by the researcher herself.

As the researcher enters the selected district, she will set appointments for data collection through the district MOHCC. This method will minimise costs associated with travel during data collection processes. Data will be collected from research participants i.e., CHWs and caregivers in the areas where they reside in.

#### Plan for data management and analysis

This will be done as follows:

Quantitative data analysis

The study variables will be related to the GMP activities done by the CHWs e.g. the number of CU5 weighed and also related to the barriers and facilitators towards GMP by caregivers. The completed questionnaires will be coded and then captured onto excel and exported to SPSS version 25.0 by the researcher. Multivariate analysis will be done followed by the normalization of the data since the study variables have different measurement units. An ordinal scale will be created and used to combine the different variables into an outcome which is the GMP index. The data file will be stored securely in a password-protected laptop and then analysed for statistical inferences i.e., multiple linear regression and comparison tests as per study objectives.

Qualitative data analysis

The method chosen for this research is Thematic analysis so as to be able to identify, organize, describe, analyse and report the themes that will be derived from this study, [6]. ATLAS.ti, the software will be used to structure and manage the data. This will involve entering the data into the software and allowing it to identify the various themes that come out from the data. The researcher will be responsible for the initial coding of the data, while the promoters will review the codes to identify the themes emerging. Through ATLAS.ti, the code-recode procedures that may take place between the researcher and promoters will be facilitated to increase the credibility and dependability of the study [1]. This data will be triangulated with findings from the quantitative results as the research seeks to answer some of the study objectives and thus contribute towards the findings of the study goal [22]. The results will be interpreted and any arising questions or lessons learnt noted down and conclusion of the study reached.

#### Data interpretation

The information from the FGDs will be *triangulated* with information from the survey questionnaires. This will ensure credibility in allowing a holistic picture of GMP activities as performed by CHWs and underpinned by the study conceptual framework, hence providing detailed and accurate answers to the research questions. Lawlor et al. [38] go on to support this strategy when they indicate that the best method in obtaining the various divergent constructions of reality that exist within a study is to obtain data on the different events and relationships from different viewpoints. Triangulation will be used because it relies on multiple forms of evidence instead of just a single incident or data point in the study.

### PHASE 3

#### GMP index development

There will be three steps in the development of the GMP index, namely: (1) variable selection, (2) examining empirical relationships of variables and combining them into an index and (3) validating the index. The index will be constructed by assigning selected variables scores. The GMP index will thus take advantage of any intensity structure that may exist among variables.

##### Variable selection

The selection of variables will be guided by a clear and precise definition of the construct itself. Face validity of the variables will also be ensured. Another important aspect to consider in the selection of variables is the degree of variation that would be provided by them. If there is no variation, it would not be very useful for the construction of an index.

##### Data synthesis and consolidation

This phase will review data analysis of the quantitative data i.e., the survey with CHWs and barrier analysis (BA) of the caregivers of CU5. The relationships among variables will be considered, with the anticipation of combining items into a single and one-dimensional construct variable. By assigning scores for particular responses on an item, a single composite index can be created through the basic summation of items. The variables whose p-values will be significant will be included in computing the index. A defined scale such as the Likert scale with at least five response categories will be used to quantify and describe the index with regards to how each valid parameter contributes to index formulation.

##### Validation of the GMP index

This stage will incorporate expert health care workers within the MOHCC as the GMP index will contribute to the classification of GMP activities in a district through the DHIS2 database. The Delphi technique will be used as a validation method to validate the GMP index. The Delphi technique is a method whose aim is to develop expert-based judgement about an epistemic question. These experts can draw on various sources of information in making their judgements such as their personal expertise and or knowledge from other types of studies [21]. Some of the typical objectives of the Delphi technique have been to develop measurement tools and identify indicators, identify the current state of knowledge on a research topic and formulating recommendations for action and prioritising measures [13], [32].

Different health care worker experts as proven by their academic and scholarly background will be purposively selected. They will be briefed on the findings of the study up to the GMP index development stage. They will be tasked with critiquing the identified parameters that will constitute the index. Their feedback will be used to make any necessary revisions to the composition of the index and thereafter guidelines of index usage will be developed.

### Ethical considerations

The following ethical considerations will be considered in this study:

#### Ethical clearance

The study proposal and tools were submitted to the Department of Public Health and the School of Public Health for quality assessment. Thereafter, were submitted to the University of Venda Human Research Ethics Committee for ethical clearance [FHS/21/PH/23/0511] and Medical Research Council of Zimbabwe ethical approval number MRCZ/A/2877.

#### Permission to conduct the study

The local level permission was sought in writing from MOHCC at the Provincial level to interview the CHWs and HCWs in Umguza district.

#### Voluntary and informed consent

To ensure that all participants voluntarily consent to participate in the study, they will be provided with a participant information letter that will explain the objective of the research. It will also be clearly explained that they have the right not to participate in the research and should not in any way feel threatened by not participating in the research [33]. If they decide to participate in the research, they will be asked to sign the informed consent form. Each participant will be allowed to refuse to participate, hence all participants should ideally be willing participants. Participants in the in-depth interviews and focus group discussions will also be given an opportunity to consent to have the interviews recorded. The researcher will explain to the participants that they can choose to have their interview recorded or not. To avoid bias created by respondents, the in-depth interviews and focus group discussions should involve only those participants genuinely willing to take part and are prepared to offer their information freely [25].

#### Privacy and confidentiality

The issue of confidentiality and privacy will be upheld in this research. It will be explained to the respondents that no respondent identifiers will be captured on field notes except for a pseudonym or a coded identifier. All information that will be recorded will be for the sole use of this research and nothing else. According to Grove, Gray and Burns [12], confidential information that is provided by the research participants must be treated as such by researchers. By participating in the research, the participants would have entrusted the researcher with a lot of private information and it is a mandate of the researcher to respect and be honourable in keeping to such principles.

#### Protection from harm

The researcher will ensure that no harm will befall the research participants as a direct or indirect consequence of the research [7]. The researcher will take the utmost care in protecting them during the process of data collection. This is protection from either physical or psychological harm or otherwise. All data collected will be kept in a locked cabinet whose access will only be by the researcher, while all typed data will be kept on a password-protected computer. This data will be stored for 5 years. The list of pseudonyms or coded identifiers that link each participant to their identifier (and in turn the participant to their interview transcript) will be securely kept by the researcher in a password protected file and computer. The participant's anonymity will also be protected when the results of the study are written up into a research report, journal article and presented to stakeholders. This will be ensured by continuing to use pseudonyms (and removing any identifying information) when presenting quotes and/or recounting the experiences of the participants.

Participants will be assured that there will be no adverse consequences to them should they decide not to participate in the study or decide not to have the interview recorded if they consent to participate in the study.

There are no foreseeable risks of injury or inconveniences which may arise from participating in this study as no form of treatment is involved. All that will be required of the participant is to answer questions that will be asked. Should such a situation arise, you will be excused from the discussion and referred to the health facility for professional counselling and care.

#### Objectivity and integrity in research

The researcher will always seek to maintain objectivity and integrity in the conduct of the research. All limits of the findings that may impair the validity of these findings will be made known [25]. In so doing, the researcher will not alter any obtained data to influence the direction of the research.

### Delimitation of the study

The study will take place in all 19 wards of Umguza district and all 186 CHWs will be interviewed. Umguza district was selected as a district of choice due to its poor performance per DHIS2 growth monitoring data.

## Plan for dissemination and implementation of results

a) The results of the study will be published in double-blinded peer-reviewed journals to share with the global audience.
b) A presentation will be made to MOHCC to share findings and proposal of adoption of GMP index in the DHIS2.
c) Meetings will be planned with the Director of Family Health and Nutrition Director within the MOHCC to plan on how MOHCC can utilise the results.
d) Study findings will be presented to UNICEF as a key donor supporting the growth monitoring programme of the MOHCC.
e) Conference presentations will be made to share the study results.
f) Presentation of study results will also be made during research days at the university
g) Meetings will be scheduled with CHWs so as to share the results of the study.

## Declaration of Competing Interest

Please **tick** the appropriate statement below (please do not delete either statement) and declare any financial interests/personal relationships which may affect your work in the box below.

The authors declare that they have no known competing financial interests or personal relationships that could have appeared to influence the work reported in this paper.

The authors declare the following financial interests/personal relationships which may be considered as potential competing interests:

Please declare any financial interests/personal relationships which may be considered as potential competing interests here.

## Data Availability

Data will be made available on request.

## Appendix Supplementary materials

Image, application 1
