# Supplementary material for: Development of a growth monitoring and promotion index to improve child health in Zimbabwe
Source: MethodsX. 2022 Dec 11;10:101958. doi: 10.1016/j.mex.2022.101958 (PMC9807990; doi:10.1016/j.mex.2022.101958)
Supplement: Supplementary file 1 [file mmc1.docx]

**Supplementary material *and/or* additional information [OPTIONAL]**

## Introduction

Globally, nearly 229 million children under three years were malnourished in 2019. Of these, more than one-quarter of all wasted children lived in Africa (UNICEF, WHO, World Bank Group, 2020). The National Nutrition survey of 2018 showed that 26,2% of children under five years in Zimbabwe are stunted. Given these statistics, it is increasingly important to monitor the growth of children to improve child nutrition and detect early any serious underlying conditions (Scherdel et al., 2016). Furthermore, growth monitoring can provide an entry point to preventive and curative health and has also been considered an integral part of programmes associated with significant reductions in malnutrition and mortality among children under five years (Pollifrone et al., 2020).

## Background

Growth monitoring (GM) as first conceptualised by Garner, Panpanich and Logan (2000) is elaborated on by Scherdel et al. (2016) as being the regular measurement of children’s weight and height, plotting the information onto a growth chart, investigating and growth abnormality. This results in the early diagnosis of serious illnesses whose prognosis can be improved through early detection. Growth monitoring is normally conducted by health workers at the health facility level and community health workers within their communities. The element of promotion through counselling by health professionals has been added to GM making it thus known as growth monitoring and promotion (GMP). This addition was done to improve the caring practices for children under five years (CU5) (Moyo & Mapulanga, 2019).

In various low-income countries worldwide, questions have been raised concerning the efficacy and cost-effectiveness of GMP. The general lack of evidence supporting existing GM practices has also been low (Scherdel et al., 2016). It is; however, important to note that GMP has the potential to alter the child undernutrition landscape as it is a preventive and promotive nutrition activity (Pollifrone et al., 2020).

Growth monitoring has been an important component of child health care in many African countries since the early 1980s, with its importance being in helping to monitor child growth (Moyo & Mapulanga, 2019). Some of the challenges faced by growth monitoring programmes have been published in the literature and occur at varying levels i.e., health facility and community level. In Ethiopia, a qualitative study revealed that some voluntary community health workers (CHWs) were reported to have insufficient skills in accurately measuring children's weight and subsequent recording on the growth chart (Bilal et al., 2014). In Zambia, the poor attendance of GMP activities has also come into question, as factors such as access to health facilities and health worker attitudes continue to affect some GMP programmes (Moyo & Mapulanga, 2019). In South Africa, Limpopo province, primary health care nurses mentioned staff shortages, work overload and lack of equipment as affecting GMP activities (Kitenge & Govender, 2013). It is these and other challenges that have affected the credibility of growth monitoring programmes worldwide. Despite some of these challenges, a study in Lesotho by Seutloali et al. (2018) that sought to explore the lived experiences of CHWs in conducting health promotion activities, noted that when CHWs take children’s weights and heights for growth monitoring, health facility nurses are able to focus on more technical tasks.

In Zimbabwe, GMP is part of the nutritional surveillance system which aims to serve as an early warning system for child growth and development problems. Acute malnutrition is monitored using the weight-for-age and mid-upper arm circumference (MUAC), while chronic malnutrition is monitored using height-for-age. The community-based growth monitoring programme is carried out at the village level by CHWs who go on to report the number of children they monitor to the health facility within their catchment area. From the health facility, this data filters up to the district as part of the district health information system (DHIS). To highlight the problem of inadequate growth monitoring practices by CHWs in Zimbabwe, a comparative process outcome evaluation by Marume et al. (2017) in Mutasa district revealed that more than 73% of children under five years in the district were missed during routine growth monitoring. Simply put, they were not weighed monthly and neither did they have their MUAC taken. Because of the extremely high number of children being missed every month, data on growth monitoring could not be meaningfully used to estimate the prevalence of acute malnutrition using the DHIS data (Marume et al., 2017), thereby reducing the usefulness of collected data for timely decision making.

Information from growth monitoring has several possible uses. The prevalence of acute malnutrition can be estimated to facilitate international comparisons and provincial variation in the prevalence of undernutrition can be used to target interventions and resources geographically within a country by the government. In addition, trends in prevalence can be examined to assess the impact of droughts or policy changes such as structural adjustments (Wright et al., 2001). Malnutrition contributes 50 to 60 percent of all deaths among children under five years, so child mortality cannot be reduced without addressing the problem of malnutrition. Growth promotion activities are essential and have the potential to achieve many important child survival interventions. Linkages between key preventive and curative health services have been created when GMP programmes have been implemented correctly (Adhikari et al., 2017). Whether there is poor or no growth monitoring, both governments and communities may also lose the conduit and driving force for delivering a package of community-based nutrition, health and poverty reduction interventions.

Besides governments not being able to intervene accordingly, where there is poor or no growth monitoring taking place in communities, CHWs and mothers end up losing a point of reference as to the growth trajectory of the child and the opportunity to intervene before a child’s condition deteriorates (Tekle et al., 2019). To illustrate the impact of GMP on child health, a comprehensive review from 1950 onwards assessing the effectiveness of child health programmes found that education on complementary feeding by CHWs at the community level was found to produce statistically significant improvements in the mean weight and height of CU5 (Freeman et al., 2017).

In Rwanda, a large assessment of CU5 in 15 districts found a reduction in the total CU5 mortality rate by 38% (p < 0,001) (Freeman et al., 2017). In the peri-urban community of Epworth in Zimbabwe, outreach work performed by CHWs facilitated the uptake of healthcare interventions and this helped mitigate the effects of health care worker shortages (Taderera, 2019). From the current Ministry of Health and Child Care (MOHCC) growth monitoring programme in Zimbabwe, Marume et al. (2017) having analysed DHIS data concluded that the growth monitoring system can potentially be used as a surveillance system that provides real-time updates on the stunting rate, thus necessitating timely interventions.

Where community GMP programmes currently exist and there is potential for improvement, it is important to maximise their potential as the impact will be related to CHW performance among many other factors (Marume et al., 2017). Some of the gaps that have been identified in the literature have been a lack of essential supplies and an unsupportive health system that demotivated CHWs thereby leading to unsatisfactory growth-monitoring practices (Pollifrone et al., 2020). The study in Zimbabwe by Marume et al. (2017), revealed that half of the CHWs had one scale each, implying that those with no scales possibly did not weigh any children monthly, while only 62% of CHWs had a MUAC tape. Despite the limitations that this study was a desk review, the authors recommended the conducting of research collecting primary data investigating further growth monitoring done by CHWs. The National Nutrition Survey of 2018 acknowledged that the provision of child health services such as growth monitoring was low and that there was a need to strengthen community-based programme delivery for communities through growth monitoring and promotion by CHWs.

In Zimbabwe, there is a paucity of research specifically looking at growth monitoring of children under five by CHWs in their villages and the barriers and facilitators towards GMP for caregivers of CU5. While the study by Marume et al. (2017) evaluated growth monitoring by CHWs, it was a desk study that only relied on secondary data thus limiting the conclusiveness of the results. Other studies have delved on the role of CHWs in Zimbabwe; Sanders (1990; 1992) focused on the development of the village health worker (VHW) programme, Gore et al. (2015) looked at the role of VHWs and challenges faced in providing primary health care (PHC), while Kambarami et al. (2016) investigated factors associated with CHW performance. Furthermore, Bilal et al. (2014) from Ethiopia also emphasise the lack of research regarding challenges in the practice of GMP at the grassroots level, among those who actually perform GMP. They highlight the need for investigations in different country contexts.

This study will seek to develop a GMP index. This index will measure both routine GMP metrics as conducted by CHWs as well as the barriers and facilitators towards GMP by caregivers of CU5. It is important to understand that both CHWs and caregivers of CU5 are crucial in ensuring the GMP of CU5 and that a comprehensive approach towards GMP should be used to improve child health. The interpretation of GMP data is currently based on a few indicators from the MOHCC DHIS2 which are heavily skewed towards data collected from CHWs, whilst leaving out the caregivers of CU5. The specific indicators in the DHIS2 are; the number of children under five weighed, number of children under five measured MUAC, (MOHCC, 2020). This approach when classifying the performance of GMP in districts has not included behaviour aspects of the caregiver thereby missing out on the importance of why caregivers of CU5 attend or not GMP activities of their children i.e., caregiver behaviours. From the literature search thus far, no such GMP index exists which combines both CHW GMP activities and caregiver behaviours and the study seeks to develop an entirely new GMP index to improve child health.

## Problem statement

Umguza district has a total of 12,675 children under five years (MOHCC,2020), 186 village health workers across 25 primary health facilities. GMP is done at the community level by CHWs and involves monthly weight and mid-upper arm circumference (MUAC) measurements, subsequent plotting on the child health card and appropriate health education/counselling for the caregiver relevant to the age of the child. Trend analysis from 2018 to August 2020 from the Umguza DHIS data shows that in 2018, the highest number of children whose weight was measured in each month was 439 (3,5%), leaving 96,5% or more children not weighed monthly. In 2019, the highest number of children whose weight was measured in a given month was 313 (2,5%), leaving 97,5% or more children not weighed each month. In 2020 so far, the highest number of children whose weight was measured in a given month is 387 (3,05%), leaving 96,95% or more children not having been weighed so far this year, (MOHCC, 2020). This also points to the fact that all the caregivers of these CU5 who are not attending GMP are being missed with regards to appropriate health education and counselling relevant to the child’s particular health condition. There have been no studies done in the district regarding the behaviour of caregivers of CU5 towards GMP activities.

These statistics point to a very big problem firstly for the children under five years as their growth is not being monitored timely. Most children are being missed in terms of their routine monthly GMP by CHWs posing a danger to their health. When GMP is either not done or is delayed, this presents challenges in child health programming. Acute malnutrition when detected early enables children to be referred to the health centres early for treatment, but if detected late, could result in child morbidity and mortality. The absence of timely and accurate information poses a challenge in the formulation of appropriate health interventions by MOHCC at the district level and at the national level. The main difficulty in interpreting the nutritional information gathered through the DHIS is that not all children are weighed by CHWs. This highlights the increased need to strengthen community-based growth monitoring in the villages, (Wright et al., 2001). Furthermore, at the national level, accurate trend analysis of growth monitoring is important in determining the impact of droughts and any other policy changes on child health and nutrition. This enables the government to plan for and allocate resources based on correct information to curb malnutrition. Investing in having accurate data from routine growth monitoring of children under five years is cheaper than having to plan for costly health and nutrition surveys to establish the prevalence of key nutrition indicators such as acute malnutrition.

Through this study, a GMP index will be developed to improve the quality of GMP activities to support the overall child health and nutrition of CU5 in the Umguza district.

## Significance of the study

This study is significant in that it might guide MOHCC on the GMP of children under five years (CU5), while also taking into account caregiver behaviours and on the other hand strengthening the performance of CHWs at the community level. It is important to understand why for years now, GMP is not being done consistently and how this can be improved and also consider caregiver behaviours towards GMP. The GMP index will be computed using data collected and will be a ward or district level index that can be added to the DHIS2 for additional GMP performance classification.

This study may benefit multiple levels of end-users. The CU5 and caregivers living in the Umguza district might benefit from health promotion activities that meet their varying needs as identified by the different behaviours related to GMP. The health centres may benefit from an easier and more informed method of classification of GMP activities in their wards as will be informed by the GMP index. The district MOHCC that manages all the CHWs in the district may be able to identify which CHWs need support and in which wards to strengthen the GMP system. They may also be able to come up with more suitable health promotion material for the varying caregiver needs.

At the national level, this study might provide a comprehensive and novel way of classifying GMP activities in the districts using the GMP index which considers both elements of CHW activities and caregiver behaviours. More appropriate CU5 interventions at the national level can be planned to make better use of resources.
